# GPR65, a novel regulator of helper T‐cell polarization in inflammatory bowel disease

**DOI:** 10.1002/ctm2.857

**Published:** 2022-06-08

**Authors:** Jessica D. Hathaway‐Schrader, Chad M. Novince

**Affiliations:** ^1^ Department of Oral Health Sciences, College of Dental Medicine Medical University of South Carolina Charleston South Carolina USA; ^2^ Department of Pediatrics‐Division of Endocrinology, College of Medicine Medical University of South Carolina Charleston South Carolina USA; ^3^ Department of Stomatology‐Division of Periodontics, College of Dental Medicine, Medical University of South Carolina Medical University of South Carolina Charleston South Carolina USA

1

The gastrointestinal tract is colonized by diverse microorganisms, harbouring the complex relationship between the host and gut microbiota. The host immune response maintains a balanced, homeostatic relationship with the gut microbiota in health. Perturbations of the indigenous gut microbiota, persistent infections, environmental factors, and genetic variants can lead to chronic immune responses and inflammatory bowel disease (IBD; i.e., Crohn's disease and ulcerative colitis).^1^ Studies have demonstrated that T cells contribute to the pathogenesis of IBD. Adoptive T‐cell transfer of naïve cells into immunocompromised mice can elicit an IBD‐like disease state.^2^ Furthermore, Crohn's disease has been linked to a T_H_1 cell response, while T_H_17 cells have been implicated in Crohn's disease and ulcerative colitis.^3^ However, the molecular underpinnings driving proinflammatory T‐cell responses in IBD are unclear.

Extracellular pH alterations activate G protein‐coupled receptors (GPRs). GPR polymorphisms are associated with autoimmune and inflammatory conditions, including atopic dermatitis, arthritis, and IBD.^4^ Of interest, GPR65 is a proton sensor expressed on diverse cell types, which has been implicated in the pathogenesis of IBD. Lin et al.^5^ found increased GPR65 in the inflamed intestinal mucosa and peripheral blood CD4^+^ T cells of patients afflicted by active IBD. In convincing and well‐designed experiments, Lin and co‐authors^5^ elucidated the role of GPR65 in CD4^+^ helper T‐cell actions contributing to IBD.

Lin and co‐authors^5^ overexpressed GPR65 in cultured human CD4^+^ T cells to show that GPR65 prompts CD4^+^ T cells to polarize into T_H_1 and T_H_17 cells. Experimentally inducing colitis in conditional GPR65‐CD4^+^ T‐cell knockout mice resulted in a milder colitis phenotype compared to control mice. Adoptive transfer of Gpr65^ΔCD4^CD45RB^high^CD4^+^ T cells into chronic colitis Rag1^–/–^ mice attenuated T_H_1 and T_H_17 cell immune responses in colonic mucosa. The elevated GPR65^+^CD4^+^ cell findings in active IBD patients and pre‐clinical study outcomes reported by Lin et al.^5^ support that GPR65 in CD4^+^ T cells acts as a positive regulator of IBD.

Prior reports relying on GPR65 global knockout mice suggest that GPR65 in epithelial cells and macrophages acts as a negative regulator of IBD.^6^, ^7^ Experimentally induced colitis in GPR65 global knockout mice resulted in an exacerbated disease phenotype compared to control mice, which led investigators to conclude that epithelial cells and macrophages lacking GPR65 caused lysosomal dysfunction and promoted intestinal inflammation.^6^, ^7^ While previous reports relied on global GPR65 knockout mice, Lin et al.^5^ employed a conditional knockout model and other approaches to show that GPR65 in CD4^+^ T cells prompts T_H_1 and T_H_17 cell polarization and exacerbates experimental IBD.

Recognizing that GPR65 actions in epithelial cells,^6^ macrophages,^7^ and CD4^+^ T cells^5^ contribute to the pathogenesis of IBD, this raises the question of whether innate immune cells regulate GPR65‐mediated T‐cell polarization towards proinflammatory T_H_1 and T_H_17 cells. Theory supports unknown intestinal antigens could lead to dysregulated proinflammatory T‐cell immune responses.^8^ However, it is unclear whether interactions between innate and adaptive immune cells within the gut are through microbe‐associated molecular pattern (MAMP) ‐ pattern recognition receptor (PRR) interactions, processing of intestinal antigens through MHC class II, or both. Investigations have reported that toll‐like receptor and MHC class II pathways can support one another due to similar processing of ligands/antigens within endosomal cellular compartments.^9^ Considering GPR65 actions maintain lysosomal pH and function in macrophages^6^ and promote T_H_1 and T_H_17 cell polarization,^5^ GPR65 could be a significant player in innate‐adaptive immune cell crosstalk during IBD.

Work by Lin et al.^5^ lays a foundation for future investigations defining GPR65's role in immune cell crosstalk, and underscores the need for research advancing knowledge about GPR65's role in gut microbiota–host interactions contributing to IBD. Because pH is a determinant for GPR65 function and IBD responses, it would be interesting to consider how shifts in the gut microbiota modulate the onset and progression of IBD. Are specific microbes or microbial consortia responsible for intestinal pH alterations that change GPR65 expression in T‐cell subsets involved gut immunosurveillance? Can these gut microbes be targeted through noninvasive interventions (i.e., dietary modifications, probiotics, prebiotics) to modulate intestinal GPR65 expression and support gut health?

Lin et al.^5^ mechanistically showed that GPR65 promotes T_H_1 and T_H_17 cell polarization and intestinal mucosal inflammation by suppressing NUAK family kinase 2 (NUAK2). This highlights opportunities for therapeutic targeting of GPR65 and NUAK2 in CD4^+^ T cells to treat IBD. Further research and development of GPR65 and NUAK2 inhibitors, improving specificity and minimizing toxicity, could lead to the efficacious targeting of CD4^+^ T cells in the treatment of IBD.^10^

## CONFLICTS OF INTEREST

The authors have declared no conflict of interest.
